# Transcriptomic profiling of rat liver samples in a comprehensive study design by RNA-Seq

**DOI:** 10.1038/sdata.2014.21

**Published:** 2014-08-26

**Authors:** Binsheng Gong, Charles Wang, Zhenqiang Su, Huixiao Hong, Jean Thierry-Mieg, Danielle Thierry-Mieg, Leming Shi, Scott S. Auerbach, Weida Tong, Joshua Xu

**Affiliations:** 1 Division of Bioinformatics and Biostatistics, National Center for Toxicological Research, Food and Drug Administration, Jefferson, Arkansas 72079, USA; 2 Center for Genomics and Division of Microbiology & Molecular Genetics, School of Medicine, Loma Linda University, Loma Linda, California 92350, USA; 3 National Center for Biotechnology Information, National Library of Medicine, National Institutes of Health, Bethesda, Maryland 20814, USA; 4 State Key Laboratory of Genetic Engineering and MOE Key Laboratory of Contemporary Anthropology, Schools of Life Sciences and Pharmacy, Fudan University, Shanghai, 201203, China; 5 Biomolecular Screening Branch, Division of the National Toxicology Program, National Institute of Environmental Health Sciences, Research Triangle Park, North Carolina 27709, USA

## Abstract

RNA-Seq provides the capability to characterize the entire transcriptome in multiple levels including gene expression, allele specific expression, alternative splicing, fusion gene detection, and etc. The US FDA-led SEQC (i.e., MAQC-III) project conducted a comprehensive study focused on the transcriptome profiling of rat liver samples treated with 27 chemicals to evaluate the utility of RNA-Seq in safety assessment and toxicity mechanism elucidation. The chemicals represented multiple chemogenomic modes of action (MOA) and exhibited varying degrees of transcriptional response. The paired-end 100 bp sequencing data were generated using Illumina HiScanSQ and/or HiSeq 2000. In addition to the core study, six animals (i.e., three aflatoxin B1 treated rats and three vehicle control rats) were sequenced three times, with two separate library preparations on two sequencing machines. This large toxicogenomics dataset can serve as a resource to characterize various aspects of transcriptomic changes (e.g., alternative splicing) that are byproduct of chemical perturbation.

## Background & Summary

Whole-transcriptome sequencing technologies, i.e., RNA-Seq, offer the capability to capture a high resolution picture of the transcriptomic landscape. With such data it is possible to evaluate the transcriptome at multiple levels including differential gene expression, allele specific expression, alternative splicing, gene fusion, and etc^1,2^. Recent studies using deep sequencing technologies have provided a more comprehensive understanding of the processes that govern the transcriptome^3^. The rapid advancement in this field is in part enabled by various collaborative efforts that have employed a crowd sourcing model to assess data utility as it pertains to challenges in different application domains. One such effort is the FDA-led MicroArray Quality Control (MAQC)^4–6,4–6,4–6^ project to assess reliability and applicability of emerging technologies including RNA-Seq in the clinical and regulatory settings.

As a part of the third phase of the MAQC project (MAQC-III), also called SEquencing Quality Control (SEQC)^6^, an RNA-Seq profiling dataset of rat liver samples was generated to assess the utility of RNA-Seq in safety assessment and toxicity mechanism elucidation. The liver was chosen because it is one of the most common target organ sites for toxicity and carcinogenicity in the rodent toxicological assessments^7^ and the regulatory community often considers mechanistic data (such as transcriptomics) in order to gauge the relevance of findings in rat liver to human health. The evaluation of such data takes place through what is call a mode of action framework. Drugs, toxicants and other environmental agents elicit toxicity in the liver by interacting with a variety of molecular targets that then elicit a complex cascade of biological processes that can either enhance or attenuate toxicity. The composite of these biological processes is referred to as a toxicological mode of action (MOA) which, as noted above, can, in part, be reflected in the agent-elicited transcriptional response. The SEQC toxicogenomics study evaluated the downstream genomic biology of chemicals representing seven MOAs in the rat liver with the purpose of characterizing the commonalities and distinctions of the different MOAs as measured by RNA-Seq and microarray.

**Figure 1:**
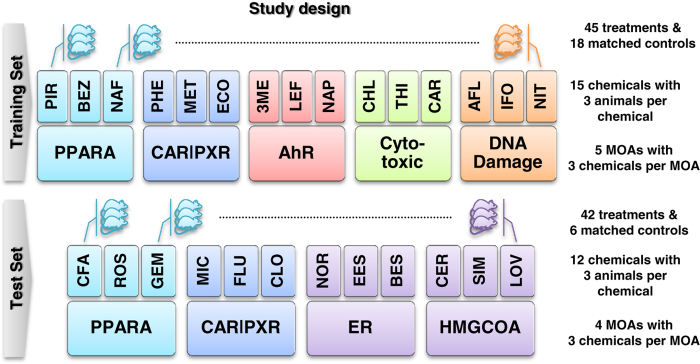
Overview of study design. This Figure was modified from Figure 1a presented in the related research manuscript. The study was comprised of a training set and a test set with the text on the right detailing the experimental design. RNA-Seq was used to profile molecular events related to treatment of rats by each chemical; each chemical is known to associate with a specific mode of action (MOA). For each MOA there were three representative chemicals and three biological replicates per chemical. The training set consisted of 15 chemicals and 5 MOAs.The test set consisted of 12 chemicals and 4 MOAs. Of the 4 MOAs in the test set two (PPARA and CAR/PXR) appeared in the training set while the other two did not.

As illustrated in Figure 1 and Table 1, RNAs from the livers of male Sprague-Dawley rats treated with one of 15 chemicals (three rats per chemical) or matched vehicle controls, were analyzed using Illumina RNA-Seq technology. This set of RNAs was referred to in the primary manuscript describing this study as the 'raining set'. These chemicals were chosen because they are expected to uniquely perturb the rat liver transcriptome at both a qualitative and quantitatively level. Sets of three chemicals share one of the five MOAs. Three MOAs are associated with well characterized receptor-mediated processes: peroxisome proliferator-activated receptor alpha (PPARA), orphan nuclear hormone receptors (CAR/PXR), and aryl hydrocarbon receptor (AhR), while the other two are non-receptor-mediated: DNA damage (DNA_Damage) and cytotoxicity (Cytotoxic). The scope and scale of this study design allowed one to observe systematic trends of various differentially expressed RNA features (mRNA, isoform, non-coding RNA, junction) alongside the degree of perturbation of rat livers and elucidate treatment-induced alternative splicing and shortening of 3′untranslated regions (UTRs). In addition to the first data set, a second, independent test dataset comprised of RNAs from the livers of male Sprague-Dawley rats treated with 12 chemicals (not used in the training set) was generated. These 12 test chemicals represent 4 MOAs, two of which are shared with the training set (CAR/PXR and PPARA) and two of which are not (estrogen receptor (ER) and HMG-CoA reductase (HMGCOA)). The inclusion of this additional set of data allowed for independent performance evaluation of RNA-Seq-based MOA classifiers generated from the training data.

In average, about 20 million paired end reads (100 bp x2) were produced for each rat liver sample from one library preparation. In addition, six samples, including three rats treated by aflatoxin B1 and three vehicle control rats, were sequenced three times, with two separate library preparations on two different Illumina sequencing machines (HiScanSQ and HiSeq 2000). These technical replicates were used to evaluate the variability introduced by different batches of library preparation and sequencing. Sample annotation, read depth, and sequencing data mapping statistics were listed in Supplementary File 1. To the best of our knowledge, this dataset represents the largest, RNA-Seq toxicogenomics dataset in the public domain. In our related work at Nature Biotechnology we provide an in depth analysis of these data and find that the cross-platform concordance in terms of differentially expressed genes (DEGs), enriched pathways, or modes of action is highly correlated with treatment effect size, gene-expression abundance, and the biological complexity of the mode of action^8^. In this Data Descriptor, we provide additional information aimed at helping others reuse and interpret these data within their own research, including more detailed methods descriptions and fuller release of ancillary datasets (e.g., the qPCR data).

## Methods

### Housing of animals

This description on the housing of animals is expanded from descriptions in the related research manuscript^8^. All biological samples employed in the studies described here were derived from the DrugMatrix tissue/RNA bank that is now owned by the National Toxicology Program (NTP). Details on the design and in life portion of these studies can be found elsewhere^9^. In short, male Sprague-Dawley [Crl:CD® (SD)|GS BR] rats [aged 6–8 weeks and weighing 200–260 g, purchased from Charles River Laboratories (Portage, MI)] were housed individually in hanging, stainless steel, wire-bottom cages in a ventilated room (temperature, 22±3 °C; humidity, 30–70%; 12 h light: 12 h dark cycle per day, 6:00 a.m.–6:00 p.m.) and received Certified Rodent Diet #5002 (PMI Feeds Inc.. Richmond, IN); chlorinated tap water was available ad libidum. Animals were acclimated for one week to the testing laboratory prior to dosing. Test chemicals were administered orally (10 ml/kg body weight in corn oil or water) or via intraperitoneal, intravenous or subcutaneous injection (5 ml/kg body weight in saline). In order to ensure a maximal transcriptional response, 5 day maximum tolerated doses (MTD) of test chemical were administered to the study animals. The MTD was determined in a 5-day range finding study in which an MTD was determined as a 5 to 10% reduction in body weight relative to control. Animals were dosed once daily for 3, 5, or 7 days, depending on the chemical, and livers were harvested 24 h after the last dose. For example, a 3 day study would start with a dose at time 0 followed by subsequent dosing at the 24 and 48 h time points. The animals would be euthanized at 72 h having received 3 repeat doses of the test chemical. Hence, animals from the 5 day studies received 5 repeated doses of chemical and animals in the 7 day studies received 7 repeated doses with each dose separated by 24 h. Animals were randomly assigned to test chemicals using a computerized body-weight stratification procedure. All toxicity studies described here were performed in a contract laboratory and therefore investigators from the sponsor were blinded to group allocation during the study. Animals were handled in accordance with United States Department of Agriculture and Code of Federal Regulations Animal Welfare Act (9 CFR Parts 1, 2, and 3). After dosing, all liver samples were harvested as 100 mg punches using six millimeter disposable biopsy punches. An appropriately staggered schedule was employed so that the harvest times were accurate to ±30 min of the designed dose-to-harvest interval.

### Sample selection

This description on sample selection is expanded from descriptions in the related research manuscript^8^. RNA samples for RNA-Seq were derived from the NTP DrugMatrix RNA bank. The following criteria were applied in the selection of treated samples: act via one of 7 modes of toxicological action, have existing Affymetrix whole genome GeneChip® Rat Genome 230 2.0 array data in the DrugMatrix Database for the same RNA sample, be derived from repeat dose toxicity studies (3, 5, or 7 days) and be from liver. Dosing level and duration for each chemical are listed in Table 1. Sets of 6 control samples were selected to match three different combinations of the route of administration (oral gavage or intraperitoneal injection) and vehicle type (nutritive or non-nutritive) that were employed for chemical administration. As with the chemical treated samples, all control samples were required to have existing Affymetrix whole genome GeneChip® Rat Genome 230 2.0 array data and to be from liver. Due to sample selection during the creation of the DrugMatrix Database Affymetrix data the paired control samples were not derived from the same animal toxicity study as the treated samples.

The samples were split into 2 sets, training and test, which were sequenced in two separate batches and run on different days. The splitting of the samples into 2 sets was done to allow for the evaluation of classifiers derived from the training data. There were 63 samples in the training set and 42 in the test set. Of the 63 samples in the training set 45 were derived from rats treated with chemicals and 18 were control samples (6 samples for each of 3 employed combinations of vehicle type and delivery route). Thirty six of the test set samples were derived from chemical treated animals and 6 were from vehicle and route matched controls. There was only one combination of vehicle type and delivery route employed in the test set. For each chemical there were three rats treated at the same dose level and there were three chemicals chosen for each MOA for the training and test set. All chemical treatments were different between the training and test set. Five MOAs (PPARA, CAR/PXR, AhR, Cytotoxic, and DNA damage) were represented in the training set and 4 MOAs (PPARA, CAR/PXR, ER, and HMGCOA) were in the test set. Two of the MOAs (PPARA and CAR/PXR) were duplicated from the test set with different test chemicals and two were without representation in the training set (Figure 1 and Table 1).

### RNA-Seq library preparation and sequencing

These descriptions are slightly expanded from descriptions in the related research manuscript^8^. Prior to library preparation, RNA quality was checked with an Agilent Bioanalyzer (Agilent Technologies, Inc.) and all of the RINs were between 8.7 and 10 except four samples with RINs between 7.4 and 7.9 (Supplementary File 1). Library preparation was performed according to the manufacture’s protocol using the Illumina TruSeq RNA Sample Preparation Kit at the Functional Genomics Core Facility at City of Hope National Medical Center. Briefly, 500 ng of each total RNA sample was mixed with 1 μl 1:100 diluted ERCC^10^ RNA spike in control mix1 or mix2 (cat 4456740, 4456739, Life Technologies, Inc.), followed by polyA mRNA selection and fragmentation, first and second strand synthesis, end repair, adenylation of 3′ ends, and index adapter ligation. Each library was subject to 15 cycles of PCR amplification, and the size distribution was examined on an Agilent Bioanalyzer (Agilent Technologies, Inc.) using a DNA 1000 chip. Each sample used one out of 12 unique indices (Illumina). All libraries displayed a band between 200–500 bp with a peak at approximately 260 bp. The libraries were quantitated with Qubit 2.0 Fluorometer (Life Technologies, Inc.), loaded at a concentration of 8.6 pM with four or six libraries pooled in one lane of Illumina V3 flow cell, and sequenced on either Illumina HiScanSQ or HiSeq 2000 Sequencing Systems with paired-end 100 bp reads.

### RNA-Seq data generation batches

Library preparation was performed in two batches with several months separating the batches. One batch contained samples in the training set and the other batch contained the test set samples. In order to assess the variation due to library preparation, six samples from the training set (three with aflatoxin B1 treatment and three matched controls) were included in the batch for the test set to track the technical reproducibility. A total of 111 libraries were constructed.

For the training set, sequencing was separated in two batches with four libraries pooled per lane, one on an Illumina HiScanSQ machine and the other on an Illumina HiSeq 2000 machine. In order to assess the variation introduced by different sequencing instruments, libraries of the six samples described above (three treated with aflatoxin B1 and three matched controls) were sequenced in both batches. For the test set, all libraries were sequenced in one batch with six libraries pooled per lane on an Illumina HiScanSQ. There were six libraries failed in a lane (due to a clogging of the fluidic system associated with that lane) that were re-sequenced with the read length reduced to paired-end 50 bp. Thus a total of 117 paired-end sequencing runs were obtained on the 111 libraries constructed from 105 RNA samples. Depths of ~23–25 million paired-end 100 bp reads were generated for each library sequencing run (Supplementary File 1). One library sequencing run was later identified as an outlier, probably due to mislabeling, and thus excluded from analysis and GEO deposition (see Technical Validation).

### RNA-Seq data processing and naming convention

After base calling, adapter trimming, and barcode demultiplexing using the sequencer manufacturer's software, sequence data with quality scores were transferred to the FDA’s National Center for Toxicological Research (NCTR) for integrity check and reformatting. A naming convention was then applied to the uniformly reformatted data with a compact digital signature (MD5 checksum) computed for each data file. The name of each data file coded the following fields with valid values in parentheses: the sequencing facility (‘COH’ for City of Hope National Medical Center & Beckman Research Institute), the facility director (‘WANG’), flow-cell ID, RNA sample ID (5 digit number), sample origin (‘L’ for liver), chemical name or control abbreviation (Table 1), vehicle type (‘NU’ or ‘NN’), delivery route (‘OG’ for oral gavage or ‘IP’ for intraperitoneal injection), barcode (six letters of A/C/G/T), lane indicator (‘s’), lane number (‘1’ –‘8’), read direction (‘1’ or ‘2’), and file type (‘fq’ for fastq). Fields were concatenated in the above mentioned order with underscores (‘_’) as the field separators. However, the data files for test set were blinded with 3-digit numbers to replace the RNA sample ID and four sample characteristic fields (i.e., sample origin, chemical, vehicle type, and delivery route) were removed. Finally, each data file was compressed individually with gzip and attached with the suffix ‘gz’. Sequence data were then duplicated and distributed immediately to data analysis teams for analysis.

## Data Records

RNA-Seq data from 116 library sequencing runs were deposited in the Gene Expression Omnibus (NCBI) under accession number GSE55347 (Data Citation 1). GSE55347 contains a processed data file listing the expression measurements for each of the rat and ERCC transcripts, which is in a tab-separated text format with the following columns: Gene_Name, Annotation_Method, NCBI_Gene_ID, RefSeq_Transcript_ID, Chromosome, Strand, Start_Base, End_Base, Gene_Description, and gene expression index for each of the 116 library sequencing runs. The complex mapping relationship between the microarray probesets and Refseq/Aceview genes was also embedded in the processed data file (‘GSE55347_TGxSEQC_GeneExpressionIndex_Magic_20120831_116samples.txt.gz’) with the first column ‘Gene_Name’ as the unique gene identifier. One gene may be mapped to multiple microarray probesets or Refseq genes. A Supplementary File was also included to explain the mapping and quantification procedure. The GEO dataset provides a link to the corresponding NCBI Sequence Read Archive (SRA) accession that contains the raw sequencing data from all sequencing sites. The corresponding microarray data were deposited in GEO under accession number GSE47875 (Data Citation 2), and qPCR data with descriptions in figshare (Data Citation 3). All SEQC (MAQC-III) data sets are available through GEO (Data Citation 4).

The table column headers for Supplementary File 1 (titled ‘Supplementary File 1-Sample Annotation and Sequencing Data Summary.xlsx’) are listed below in their sequential order:

RNA ID,

Sample Description,

RNA-Seq Run ID,

Animal ID,

Affy_ID (Microarray),

Chemical Name,

Chemical Name Abbreviation,

Vehicle Type,

Route,

Duration (days),

Dose (mg/kg/day),

Mode of action (MOA),

Original Set Annotation,

RNA Extraction Date,

Microarray Hybridization Date,

RNA-Seq Run Date,

RIN Measured Before RNA-Seq Library Preparation,

ERCC_Mix Spike-in,

Machine,

Sequencing_Lab,

Sequencing_Lane,

Number_of_Reads,

Read_Length (nt),

GC_Content_in_the_reads,

%Reads_Aligned,

Number of Aceview genes expressed (with at least 4 reads mapped),

Average insert length measured after alignment (bp),

Number of mismatches per kilobase aligned,

%Reads aligned to ERCC transcripts,

%Reads aligned to rat rRNA,

%Reads aligned to rat mitochondria,

%Reads aligned to rat RefSeq genes,

%Reads aligned to rat genome,

fastq file_name_1,

fastq file_name_2.

## Technical Validation

**Figure 2:**
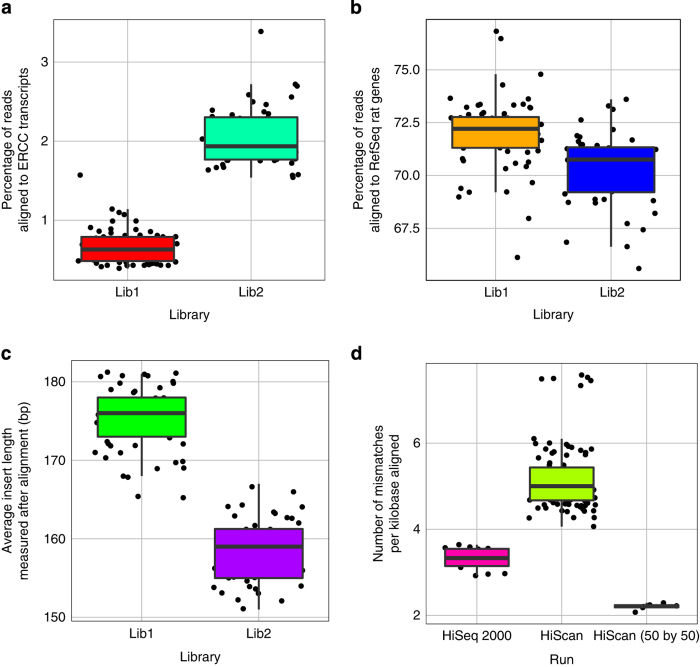
Trends of quality metrics per library preparation and sequencing run batch. Each subpanel illustrates the results for one quality metric: (**a**) percentage of reads aligned to ERCC transcripts, (**b**) percentage of reads aligned to RefSeq rat genes, (**c**) average insert length in base pairs measured after alignment, and (**d**) average number of mismatches per kilobases aligned. Each box plot displays the range and distribution of a quality metric computed for sequencing sample runs in the library preparation or sequencing batch. The horizontal line is the median, the top of the box is the upper quartile, and the bottom of the box is the lower quartile.

The first aim of QC and validation was to assess the variation introduced by library preparation and sequencing batches by taking advantage of the ERCC spike-ins and technical replicates built into different batches. ERCC mixes 1 and 2 were spiked in to RNA samples prior to library preparation. After aligning the reads to ERCC transcripts, RefSeq rat genes, and rat genome, we chose four different quality metrics including percentage of reads aligned to ERCC transcripts, percentage of reads aligned to RefSeq rat genes, average insert length measured after alignment, and base error rate measured after alignment. As shown in Figure 2a, the ERCC spike-in ratios were quite different between two batches of library preparation. This difference was also consistent with the difference in the percentage of reads aligned to RefSeq rat genes (Figure 2b). Thus the expression quantification for rat genes should be normalized by the total reads mapped to rat genes to account for the difference in ERCC spike-in ratio. The average insert length was apparently different between the two library preparation batches (Figure 2c). Its effect on gene expression quantification has not been extensively studied^11^. Since the relative change was about 10%, we assumed the effect may not be pronounced. The sequence error rates were plotted in Figure 2d, which were influenced by a number of factors including read length, sequencing instruments, and lane to lane variation. Not surprisingly, the shorter (paired-end 50 bp) reads led to the lowest error rates^12^. The HiSeq 2000 machine performed better than both HiScanSQ machines. A technical glitch associated with a specific lane caused higher error rates for six libraries sequenced on a HiScanSQ machine.

**Figure 3:**
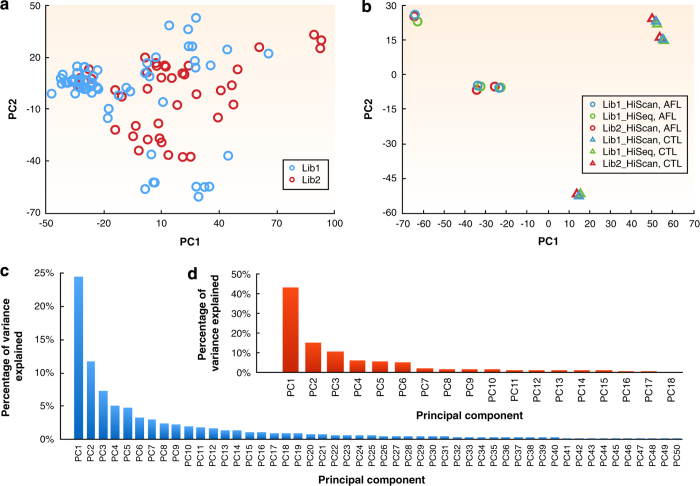
Principle component analysis (PCA) to assess the library preparation batch effect. Each sample is denoted by a circle or triangle. (**a**) A 2D PCA plot of all samples shows no separation between the two library preparation batches, i.e., Lib1 and Lib2. The analysis was done with the top 10,000 genes selected by average expression level ranking. (**b**) A 2D PCA plot of all 3 technical replicas for each of the six samples (3 treated by aflatoxin B1 (AFL) and 3 matched controls (CTL)). (**c**,**d**) Two bar charts plot the percentage of variance explained by each of the top principle components for the corresponding PCA plots (**a**,**b**).

Since the quality metrics showed the difference introduced by library preparation and sequencing run batches, their effects on gene expression quantification should be evaluated. Utilizing the technical replicas built into the sequencing experiment, we computed the correlation of expression levels between technical replicas for each of the six samples (3 treated by aflatoxin B1 and 3 matched controls) and found out that the expression levels were highly correlated (R^2^ greater than 0.98 for each sample). According to principle component analysis (PCA) of these six samples (Figure 3b), the variation introduced by library preparation and sequencing was much smaller than the difference among biological replicates. There was also a visible separation between the treated and control groups along the first principle component (PC1 on the x-axis), which explained over 40% of variance (Figure 3d). A similar PCA plot of all samples showed no separation of samples between two library preparation batches (Figure 3a), with the first two principle components explaining about 40% of variance (Figure 3c). In summary, the library preparation batch effect is negligible and this RNA-Seq dataset exhibits high reproducibility and quality.

**Figure 4:**
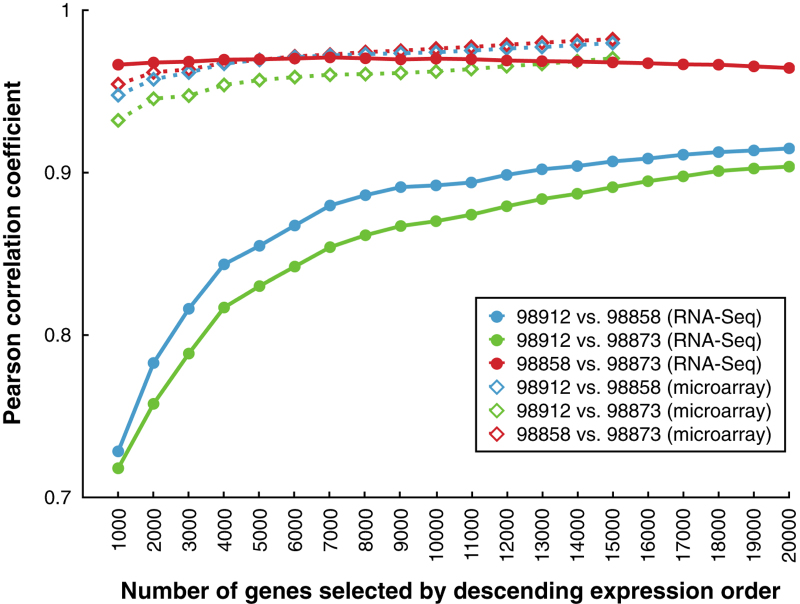
Pairwise Pearson correlation coefficients for samples treated by carbon tetrachloride (CAR). The x-axis lists the number of genes selected by descending expression order. The y-axis is Pearson correlation coefficient. Results are plotted with hollow diamonds for microarray and solid circles for RNA-Seq. For RNA-Seq, the unexpected low correlation coefficients involving RNA sample 98912 revealed its dissimilarity to other two samples and thus identified it as an outlier.

Finally, we computed the correlation of RNA-Seq expression levels between samples treated by the same chemical and compared the results with the corresponding ones calculated from the microarray data. The calculation of Pearson correlation was repeated multiple times and plotted with different numbers of genes selected by descending expression order (Figure 4). The results should be consistent between these two gene expression profiling technologies given their largely compatible performance reported in the literature^13,14^. Through this QC procedure, one sample, supposedly treated by carbon tetrachloride (CAR) with RNA_ID ‘98912’, was discovered as an outliner, probably due to mislabeling. The sample was then excluded from data deposition and any further analysis.

## Usage Notes

Many publicly available software packages, e.g., Bowtie^15^, TopHat^16^, and Cufflinks^17^, could be used for mapping and quantification of RNA-Seq data. Several reference genomes of rat could be used as the mapping template, such as RefSeq, UCSC, Aceview and Ensembl. Many publicly available software packages, e.g., limma^18^, edgeR^19^, and DESeq^20^, could be used for differential gene expression analysis of the read count data. Six pipelines were built for mapping and quantification of RNA-Seq data and documented in our related research manuscript^8^.

The three technical replicates were included for each of three samples treated with aflatoxin B1 and three matched controls, with the same library run on different sequencing machines or separate libraries construction derived from the same sample. These replicate library/sequencing runs could be used for library preparation and sequencing platform quality control or as super deep sequence data for aflatoxin B1 study by merging the reads for each sample from all the sequencing runs.

## Additional information

**How to cite this article:** Gong, B. *et al.* Transcriptomic profiling of rat liver samples in a comprehensive study design by RNA-Seq. *Sci. Data* 1:140021 doi: 10.1038/sdata.2014.21 (2014).

## Supplementary Material



Supplementary Table 1

## Figures and Tables

**Table 1:** Modes of action and exposure of the chemicals used in the study

**SET**	**MIE/MOA**	**Chemical Name**	**Abbreviation**	**Dose (mg/kg/day)**	**Duration (days)**	**Structure Activity Group; Therapeutic indication**
TRAINING	AHR	3-METHYLCHOLANTHRENE	3ME	300	5	Toxicant, Ah receptor agonist, DNA alkylator
TRAINING	AHR	BETA-NAPHTHOFLAVONE	NAP	1500	5	Toxicant, Ah receptor agonist
TRAINING	AHR	LEFLUNOMIDE	LEF	60	5	Inhibits pyrimidine /purine metabolism, dihydroorotase inhibitor; Antirheumatic Disease Modifying Agent
TRAINING	CAR/PXR	ECONAZOLE	ECO	334	5	Sterol 14-demethylase inhibitor, fluconazole like; Antifungal azole
TRAINING	CAR/PXR	METHIMAZOLE	MET	100	3	Thyroperoxidase inhibitor; Thyroid and Antithyroid Agent
TRAINING	CAR/PXR	PHENOBARBITAL	PHE	54	5	GABA agonist; Antiepileptics / Anticonvulsants
TRAINING	CYTOTOX	CARBON TETRACHLORIDE	CAR	1175	7	Toxicant, free radical generator
TRAINING	CYTOTOX	CHLOROFORM	CHL	600	5	Toxicant, free radical generator
TRAINING	CYTOTOX	THIOACETAMIDE	THI	200	5	Toxicant, free radical generator
TRAINING	DNA DAMAGE	AFLATOXIN B1	AFL	0.3	5	Toxicant, DNA alkylator
TRAINING	DNA DAMAGE	IFOSFAMIDE	IFO	143	3	DNA-alkylator, nitrogen mustard; Antineoplastic
TRAINING	DNA DAMAGE	N-NITROSODIMETHYLAMINE	NIT	10	5	Toxicant, DNA alkylator
TRAINING	PPARA	BEZAFIBRATE	BEZ	617	7	Peroxisome proliferator; Hypolipidemic Agent
TRAINING	PPARA	NAFENOPIN	NAF	338	5	Peroxisome proliferator; Hypolipidemic Agent
TRAINING	PPARA	PIRINIXIC ACID	PIR	364	5	Peroxisome proliferator; Hypolipidemic Agent
TEST SET	CAR/PXR	CLOTRIMAZOLE	CLO	89	5	Sterol 14-demethylase inhibitor; Antifungal azole
TEST SET	CAR/PXR	FLUCONAZOLE	FLU	394	5	Sterol 14-demethylase inhibitor, fluconazole like; Antifungal azole
TEST SET	CAR/PXR	MICONAZOLE	MIC	920	5	Sterol 14-demethylase inhibitor, fluconazole like; Antifungal azole
TEST SET	ER	BETA-ESTRADIOL	BES	150	5	Estrogen receptor agonist, steroidal; Bone Mineral Homeostasis
TEST SET	ER	ETHINYLESTRADIOL	EES	10	5	Estrogen receptor agonist, steroidal; Hormone replacement
TEST SET	ER	NORETHINDRONE	NOR	375	5	Progesterone receptor agonist; Ovulation inhibitor
TEST SET	HMGCOA	CERIVASTATIN	CER	7	5	HMG-CoA reductase inhibitor; Hypolipidemic Agent
TEST SET	HMGCOA	LOVASTATIN	LOV	450	5	HMG-CoA reductase inhibitor, non-aromatic; Hypolipidemic Agent
TEST SET	HMGCOA	SIMVASTATIN	SIM	1200	3	HMG-CoA reductase inhibitor, non-aromatic; Hypolipidemic Agent
TEST SET	PPARA	CLOFIBRIC ACID	CFA	448	5	PPAR alpha agonist, fibric acid; Hypolipidemic Agent
TEST SET	PPARA	GEMFIBROZIL	GEM	700	7	PPAR alpha agonist, fibric acid; Hypolipidemic Agent
TEST SET	PPARA	ROSIGLITAZONE	ROS	1800	5	PPAR gamma agonist, thiazolidinedione, antidiabetic
MOA denotes the mode of action. MIE denotes the molecular initiating event. Dose unit is mg/kg/day and the unit for duration is day.						
This table is taken from Supplementary Table 2 in the related research manuscript.						
